# A deletion in the intergenic region upstream of *Ednrb* causes head spot in the rat strain KFRS4/Kyo

**DOI:** 10.1186/s12863-017-0497-3

**Published:** 2017-03-29

**Authors:** Minako Yoshihara, Tetsuya Sato, Daisuke Saito, Osamu Ohara, Takashi Kuramoto, Mikita Suyama

**Affiliations:** 1 Medical Institute of Bioregulation, Kyushu University, Fukuoka, 812-8582 Japan; 2 AMED-CREST, Japan Agency for Medical Research and Development, Fukuoka, 812-8582 Japan; 3 Department of Technology Development, Kazusa DNA Research Institute, Kisarazu, Chiba, 292-0818 Japan; 4 Institute of Laboratory Animals, Graduate School of Medicine, Kyoto University, Kyoto, 606-8501 Japan

**Keywords:** Target capture sequencing, Exome, Conserved non-coding sequence, Regulatory mutation, Comparative genomics, Rat, Head spot

## Abstract

**Background:**

Head spot is one of the phenotypes identified in the KFRS4/Kyo rat strain. Although previous linkage analysis suggested that *Ednrb*, which is frequently involved in coat color variations in various animals, could be the gene responsible for this phenotype, no mutations have been identified in its coding region.

**Results:**

To identify mutations causative of this phenotype in KFRS4/Kyo, we analyzed target capture sequencing data that we recently generated. Our target capture method has a unique feature, i.e., it covers not only exonic regions but also conserved non-coding sequences (CNSs) among vertebrates; therefore, it has the potential to detect regulatory mutations. We identified a deletion of approximately 50 kb in length approximately 50 kb upstream of *Ednrb*. A comparative analysis with the epigenomic data in the corresponding region in humans and mice showed that one of the CNSs might be an enhancer. Further comparison with Hi-C data, which provide information about chromosome conformation, indicated that the putative enhancer is spatially close to the promoter of *Ednrb*, suggesting that it acts as an enhancer of *Ednrb*.

**Conclusions:**

These *in silico* data analyses strongly suggest that the identified deletion in the intergenic region upstream of *Ednrb*, which might contain a melanocyte-specific enhancer, is the mutation causative of the head spot phenotype in the KFRS4/Kyo rat strain.

**Electronic supplementary material:**

The online version of this article (doi:10.1186/s12863-017-0497-3) contains supplementary material, which is available to authorized users.

## Background

Fancy rat colonies carry various mutations, such as those for coat color and pattern [1]. These are among the most prominent phenotypes often observed in other domesticated animals, such as dogs, cats, horses, and mice. KFRS4/Kyo is an inbred rat strain derived from the crossing of a fancy rat with the PVG/Seac rat strain, which possesses various phenotypes, including white spotting on the head (head spot; *hs*) and abnormal outer ear morphology (dumbo; *dmbo*), both of which were shown to be autosomal recessive phenotypes [1]. Although a mutation causative of the dumbo phenotype was successfully identified by fine-scale linkage analysis [1] followed by genome resequencing [2], the mutation causative of the head spot phenotype still remains to be identified [1]. To date, a linkage analysis has been performed on the head spot phenotype, and the mutation responsible for it was shown to reside in chr15:84.6–91.2 Mb (rat rn4 assembly) [1].

Several genes involved in coat color variations have been identified [3]. *Ednrb* is one such gene responsible for distinctive coat color phenotypes in various species, for example, Waardenburg syndrome type 4 (WS4) in humans [4], aganglionosis in rats [5–7], piebald-lethal in mice [8], Lethal White Foal Syndrome in horses [9], and panda plumage color phenotype in Japanese quails [10], some of these species possess a characteristic head spot. In the case of the head spot phenotype in KFRS4/Kyo, *Ednrb* was suspected to be the gene responsible for this phenotype because it is located within the genomic interval identified by the linkage analysis. However, no mutations were found in the gene body of *Ednrb* in KFRS4/Kyo [1]. One possible explanation for these results is the existence of a mutation associated with *Ednrb* gene regulation somewhere around *Ednrb* in the interval identified by the linkage analysis.

There are several approaches to identify a gene responsible for a certain phenotype. These include linkage analysis, genome-wide association study, and exome sequencing. Among these methods, exome sequencing has the potential to directly identify a mutation causative of a phenotype, and has been widely used in the field of genomics of diseases since its first application to identify a mutation responsible for a genetic disorder [11]. Although exome sequencing has been successfully applied to identify mutations causative of many genetic disorders (for a review, see [12]), it has a drawback in that it can only identify mutations in the target regions, namely, annotated exons and the neighboring sequences, and it cannot, in principle, detect causative mutations if they are located in deep intergenic or intronic regions. To partly overcome this drawback while maintaining efficiency in terms of not sequencing genomic regions with lower selective constraints, we recently devised a novel target capture sequencing method, TargetEC, which can detect mutations not only in exonic parts of genes but also in conserved non-coding sequences that are thought to be important for gene regulation [13].

In this study, we analyzed our data obtained by the TargetEC method [13] to identify a mutation causative of head spot in KFRS4/Kyo rats, and found a candidate deletion in the intergenic region upstream of *Ednrb*. We utilized the data from a linkage analysis, as well as *in silico* comparative analyses of epigenomic data obtained from international consortia [14, 15] and chromosome conformation data obtained from Hi-C analysis of human and mouse cell lines [16, 17], to obtain evidence supportive of our hypothesis that this deletion might contain a melanocyte-specific enhancer of *Ednrb*.

## Methods

### Sequence reads

We used the sequence read data of the KFRS4/Kyo inbred strain generated by the TargetEC method, which is a target capture method focusing only on exons and conserved non-coding sequences (CNSs), obtained from our previous study (DDBJ Sequence Read Archive accession number: DRA004543) [13]. In conventional exome sequencing, the total length of the target regions is about 50 Mb, whereas in the TargetEC method, which also covers CNSs, the total length of the target regions is about 150 Mb and they are scattered over the chromosomes. The sequence read data obtained by the TargetEC method applied to the PVG/Seac inbred strain (accession number: DRA004543) [13], which was crossed with a fancy rat to establish the KRFS4/Kyo inbred strain [1] and does not possess head spot phenotype, was used as a control. The sequence read data obtained by the TargetEC method applied to other 20 rat inbred strains (BDIX/NemOda, BDIX.Cg-Tal/NemOda, BN/SsNSlc, BUF/MNa, DOB/Oda, F344/DuCrlCrlj, F344/Jcl, F344/NSlc, F344/Stm, HTX/Kyo, HWY/Slc, IS/Kyo, IS-*Tlk*/Kyo, KFRS3B/Kyo, LE/Stm, LEC/Tj, NIG-III/Hok, RCS/Kyo, ZF, ZFDM) (accession number: DRA004543) [18], non of which possess head spot phenotype, was used to filter common variants.

### Quality control of the sequencing reads and mapping

A detailed description of the analysis of the sequence reads is available elsewhere [13]. In brief, the reads generated using the Illumina HiSeq platform were subjected to quality control to exclude low-quality reads. We used the FastQC program (http://www.bioinformatics.babraham.ac.uk/projects/fastqc/) to evaluate the overall read quality. We trimmed reads from the 3′-end to address low-quality bases (quality score of <30). After this trimming step, shortened reads (length of <30) were discarded from further analysis. Then, the reads were mapped to the rat genome assembly rn5 (RGSC 5.0, March 2012), which was downloaded from the UCSC Genome Browser [19], by using BWA (v0.7.4) [20] with the default parameters. SAMtools (v0.1.12a) [21], Picard tools (v1.87) (http://broadinstitute.github.io/picard/), and the Genome Analysis Toolkit (GATK; v3.6) [22] were used for post-processing of the mapped reads [13].

### Identification and annotation of variants

Variant calling was conducted using the HaplotypeCaller utility in GATK. ANNOVAR (version 22-March-2015) [23] was used for the functional annotation of variants, with RefSeq genes and Ensembl genes as the rat gene annotations.

### Genome data analysis

The Integrative Genomics Viewer (IGV; v2.2.13) [24] was used to visualize the mapped reads and variants. The UCSC Genome Browser was used for the comparative analysis of genomes and their associated annotations [19]. Spatial proximity on the chromosome was analyzed using the ChromContact web server [17], which uses high-resolution Hi-C data for human cell lines [16].

## Results

### Identification of a candidate mutation for the head spot phenotype in KFRS4/Kyo

**Fig. 1:**
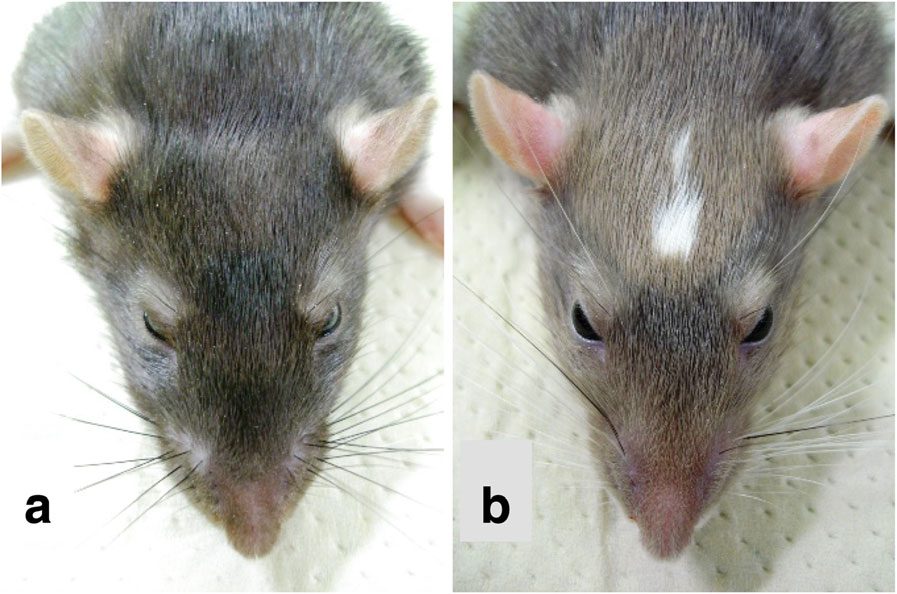
Photographs of the head spot phenotype compared with a normal control. (**a**) The control PVG/Seac strain and (**b**) the KFRS4/Kyo strain, which shows the head spot phenotype

To identify a candidate mutation for the head spot phenotype in KFRS4/Kyo (Fig. 1), first we analyzed the data obtained by the TargetEC method, which is designed to specifically capture and sequence the genomic regions corresponding to exons and CNSs in rats (DDBJ Sequence Read Archive accession number: DRA004543) [13]. Among 134,604 single nucleotide variants (SNVs) and 25,578 indels identified by TargetEC, we focused only on those mutations found within the genomic interval identified by a linkage analysis (chr15:84.6–91.2 Mb in the rn4 assembly, which corresponds to chr15:89.0–94.8 Mb in the rn5 assembly) [1]. This narrowed down the number of mutations to 211 SNVs and 58 indels.

First, we compared the 211 SNVs with those identified in the PVG/Seac inbred strain [13], which is the one used to establish the KFRS4/Kyo strain by crossing with a fancy rat [1], and in other 20 inbred rat strains (BDIX/NemOda, BDIX.Cg-Tal/NemOda, BN/SsNSlc, BUF/MNa, DOB/Oda, F344/DuCrlCrlj, F344/Jcl, F344/NSlc, F344/Stm, HTX/Kyo, HWY/Slc, IS/Kyo, IS-*Tlk*/Kyo, KFRS3B/Kyo, LE/Stm, LEC/Tj, NIG-III/Hok, RCS/Kyo, ZF, ZFDM) [18]. None of these 21 strains possess head spot phenotype. From the 211 SNVs, we filtered out those SNVs that were commonly observed in at least one of the 21 inbred strains. This filtering drastically reduced the candidate SNVs, and only one SNV remained as a KFRS4/Kyo-specific SNV (Additional file 1: Table S1). This SNV (chr15: 93,740,390 A → G) does not seem to be a causative mutation for the phenotype, because it is located in an intergenic region with low conservation among vertebrates (phastCons score = 0.000). Although the TargetEC method is designed to capture exons and CNSs, such region can be a target because a shorter region must be expanded to at least 100 bp in capture probe design to ensure efficient capture of the region [13]. Next, we compared the 58 indels with those identified in the 21 strains. After filtering out the commonly observed indels, 10 indels remained as KFRS4/Kyo-specific indels (Additional file 1: Table S1). All of these indels do not seem to be causative mutations for the phenotype, because they are located in intergenic regions with either low conservation or indels also in other species. As reported previously [1], and also confirmed in this study, there were no KFRS4/Kyo-specific SNVs or indels on *Ednrb*, one of the genes residing within the genomic interval identified by the linkage analysis and suspected to be a candidate gene for its involvement in the particular coat color phenotypes observed in other animals [8, 9] as well as in rats [5–7].

**Fig. 2:**
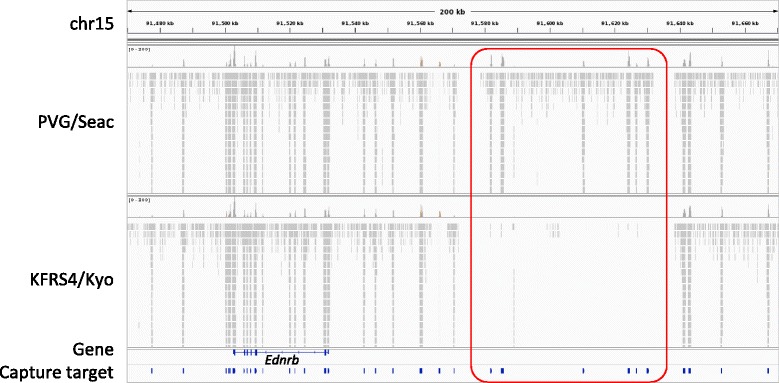
A deletion found upstream of *Ednrb* in KFRS4/Kyo. This plot was created using Integrative Genomics Viewer [24]. The genomic interval of chr15:91,470,000–91,670,000 is shown. The upper track shows the mapped reads obtained from the control PVG/Seac strain, and the lower track shows the mapped reads obtained from the KFRS4/Kyo strain. The gene track and the track for the capture target regions are indicated below the tracks of the mapped reads. The deletion found in KFRS4/Kyo is indicated by a red rectangle

Since we could not detect any significant SNVs or indels in the regions covered by the TargetEC method within the genomic interval identified by the linkage analysis [1], we next sought large deletions of non-coding sequences that might act as elements regulating gene expression. To identify such deletions, we applied the MACS2 program [25], a peak-calling software originally developed for ChIP-seq experiments, to the mapping data of the reads obtained by the TargetEC method for each strain. By comparing with the mapping data obtained from the PVG/Seac strain, which does not possess the head spot phenotype, we detected a deletion of approximately 50 kb in length located approximately 50 kb upstream of *Ednrb* in the KFRS4/Kyo strain (Fig. 2). Other than *Ednrb*, there are nine genes in the genomic interval identified by the linkage analysis, but these genes are located more than 500 kb apart from the deletion and seem not to be responsible for the head spot. The deletion is comprised of six consecutive target regions of the TargetEC method. We confirmed the deletion by PCR experiments (Additional file 2: Figure S1). We compared the locus of the deletion to the peak-calling results for the other 20 inbred rat strains [18], and found that this is the only deletion specifically found in the KFRS4/Kyo strain (Additional file 3: Figure S2) in the genomic interval identified by the linkage analysis [1] and hence thought to be a candidate mutation for the head spot phenotype in KFRS4/Kyo.

### Epigenetic features in the deleted genomic region

**Fig. 3:**
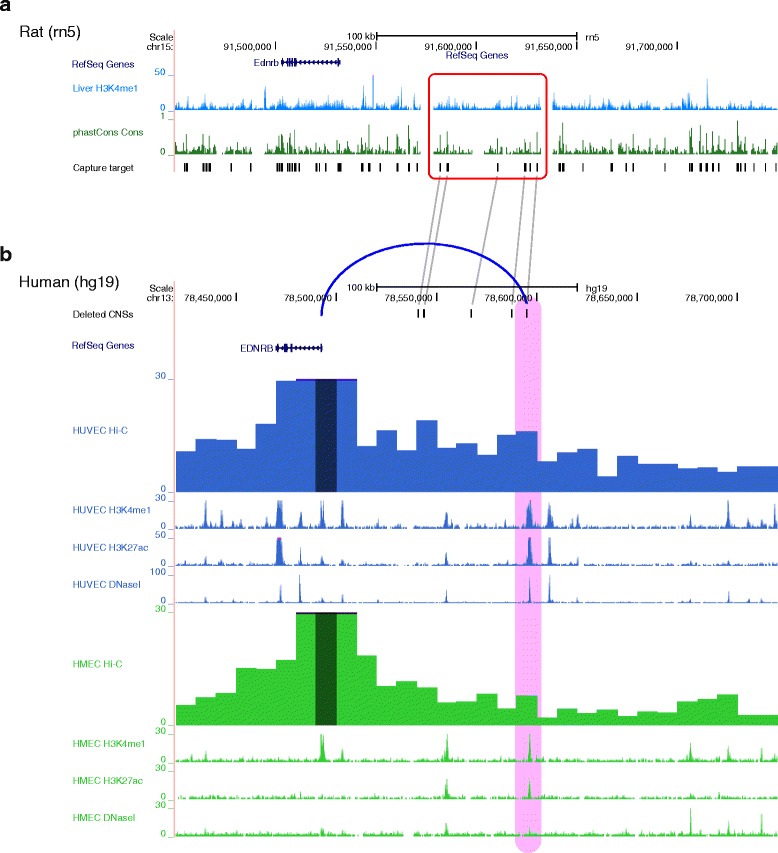
Genome browser views of the upstream region of *Ednrb*. (**a**) The genomic region in rats (*rn5*). Four tracks are shown (*from the top*): (i) RefSeq genes; (ii) the profile of H3K4me1 for the rat liver; (iii) the phastCons conservation score; (iv) the capture target regions. The deletion found upstream of *Ednrb* in KFRS4/Kyo is indicated by a red rectangle. (**b**) The corresponding genomic region in humans (*hg19*). Four groups of tracks are shown (*from the top*): (i) the positions that correspond to the deleted CNSs in rat; (ii) RefSeq genes (*only the orthologous gene between human and rat is shown*); (iii) the contact profile generated from Hi-C data of human umbilical vein endothelial cells (*HUVEC*) and the profiles of H3K4me1, H3K27ac, and DNase I hypersensitive sites for HUVEC; (iv) the contact profile generated from Hi-C data of human mammary epithelial cells (*HMEC*) and the profiles of H3K4me1, H3K27ac, and DNase I hypersensitive sites for HMEC. The corresponding CNSs that are deleted in rats are connected by gray lines. The anchor position, which contains the transcription start site of *Ednrb*, is indicated by a dark-colored highlight in the contact profile. The region containing a predicted enhancer is highlighted in pink. A possible three-dimensional interaction between the enhancer and the promoter of *Ednrb* is indicated by a blue arc

Based on the distance between the deletion and *Ednrb* gene, and also from the head spot phenotype, we hypothesized that the deleted region might contain a melanocyte-specific distant enhancer of *Ednrb*. To obtain evidence supportive of this hypothesis, we analyzed epigenomic data obtained from various sources. Although epigenomic data is rather limited for rat comparing to those for human or mouse, there is a data for H3K4me1, one of the representative histone modification for enhancers, of rat liver [26]. From the profile of this histone modification, it seems that some of the deleted probes might work as enhancers (Fig. 3a). To further obtain supportive evidence, we analyzed epigenomic data collected by the ENCODE Project [14]. Via recent progress in the accumulation of epigenomic data, there is already substantial information about the genome-wide distribution of enhancer-specific histone modifications, namely, H3K4me1 and H3K27ac, and transcription factor binding sites identified as DNase I hypersensitive sites [14] in various cell types, especially in human. To compare epigenomic profiles between different species, we converted the genomic coordinates of the deletion in rats to the corresponding coordinates in human, and analyzed the epigenetic data for that region. It is shown that the epigenomic features are, to some extent, conserved among different species [27], so it is reasonable to make use of the epigenomic data obtained for other species in which substantial data are available. We used the LiftOver function in the UCSC Genome Browser for this conversion of the genomic coordinates [28].

For the deleted region, the corresponding region in humans was found at chr13:78.5–78.6 Mb (hg19 assembly) (Fig. 3). The sequence identity in this region between rat and human genomes is 38.5%. The relative position and orientation of *Ednrb* are also similar between rats and humans. Because the CNSs in rat, for which we designed the probes for the TargetEC method, are highly conserved among vertebrates, five of the six CNSs in the deleted sequence have their counterparts in the syntenic region in human. The sequence identity for each of the CNSs between rat and human genomes ranges from 95.2 to 99.8%. From the epigenetic features, we found two sequence regions that seem to be enhancers, characterized by prominent peaks of H3K4me1, H3K27ac, and DNase I hypersensitive sites, within the region that corresponds to the deleted region in KFRS4/Kyo. One of the putative enhancers is located in the corresponding CNSs in rat, indicating that it might also work as an enhancer in rat.

### Possible three-dimensional (3D) interaction between the candidate enhancer and the promoter of *Ednrb*

To further confirm whether some of the putative enhancers identified in the deleted CNSs correspond to distant enhancers of *Ednrb*, we analyzed possible 3D interaction of the chromosome between the putative enhancers and the transcription start site (TSS) of *Ednrb*. We used Hi-C data for this analysis. Hi-C is a method for studying 3D chromatin interactions by cross-linking spatially proximal genomic fragments followed by high-throughput paired-end DNA sequencing [29]. It has been successfully applied to identify long-range enhancer-promoter interactions [16]. Although Hi-C data for rats are not yet available, we transferred the genomic coordinates for rats to those for humans and inferred the 3D interactions in rats because it has been shown that overall topology does not differ significantly between distinct cell types and even between species [30, 31]. By using the ChromContact web server [17], which visualizes Hi-C data in a 4C-like representation by specifying a position of interest as an anchor, we plotted a contact profile of spatial proximity to the TSS of *Ednrb* (Fig. 3). The resolution of Hi-C experiments has improved to a maximum of the kilobase order in recent years [16], and we used a 10-kb resolution in the present study. As shown in the contact profile, the putative enhancer at the CNS is located within a peak of the contact profile, indicating a possible interaction between TSS of *Ednrb* (i.e., anchor) and the putative enhancer, although the distance between them in terms of the genomic sequence is approximately 100 kb. This further supports the hypothesis that the putative enhancer in the deleted genomic region might be a melanocyte-specific enhancer of *Ednrb*.

We also analyzed the epigenomic features of the corresponding genomic region in mouse by using the Virtual 4C web application (http://promoter.bx.psu.edu/hi-c/virtual4c.php) (Additional file 4: Figure S3). We used the data for CH12 cell line, which is derived from B-cell lymphoma, because this is the only cell line with high resolution Hi-C data (5 kb resolution) in mouse. Although the Hi-C signal is only weakly support the interaction between the predicted enhancer and the promoter of *Ednrb*, DHS Linkage [32], which is the prediction of the interaction between a pair of genomic regions based on the correlation of DNase I hypersensitivity site data from various cell types, indicates that the predicted enhancer might interact with the promoter of *Ednrb*. A peak for H3K4me1 is also assigned to the predicted enhancer in CH12 cell line. These data in mouse further support that the deleted genomic region might act as an enhancer of *Ednrb*.

## Discussion

In the present study, we identified a deletion that is predicted to be causative of the head spot phenotype of the KFRS4/Kyo rat strain in the genomic interval determined by linkage analysis for this phenotype [1]. This deletion is located upstream of *Ednrb*, one of the genes responsible for Waardenburg syndrome type 4 (WS4), also known as Waardenburg-Shah syndrome, characterized by hypopigmentation of hair, skin, and eyes, congenital sensorineural deafness, and Hirschsprung disease, which exhibits aganglionic megacolon [4]. The involvement of *Ednrb* in WS4 is explained by its function in the development and migration of enteric neurons and melanocytes, both of which originate from neural crest cells. It has been revealed that SOX10 acts as an upstream transcription factor of *Ednrb* in the development of enteric neurons [33]; however, it seems that SOX10 is not involved in the regulation of *Ednrb* expression during melanocyte development [33, 34] and it is suggested that there might be other upstream factors of *Ednrb* expression for the development and migration of melanocytes [33, 35]. The KFRS4/Kyo strain possesses a head spot phenotype, but not aganglionic megacolon. This suggests that the head spot phenotype observed in KFRS4/Kyo is not due to a defect in the gene body of *Ednrb*, but is caused by a mutation in a regulatory region specific for the differentiation and migration of melanocytes. Indeed, the gene structure of *Ednrb* itself does not have any mutations. From these observations, it is strongly suggested that the deletion identified upstream of *Ednrb* might contain a melanocyte-specific enhancer that is responsible for the head spot phenotype of KFRS4/Kyo.

Although the lines of evidence presented in this study strongly suggest that the deletion found upstream of *Ednrb* contains a melanocyte-specific enhancer of the gene, direct experimental proof of this is still required to conclusively show its functional involvement. One possibility for obtaining such proof is qRT-PCR to quantify the difference in expression level between the wild type and the mutant strains. However, it will be difficult to detect any significant difference in the *Ednrb* expression level because the head spot phenotype is focal to a specific area. Moreover, EDNRB signaling is thought to be required only in a certain period in the early developmental stage, in which neural crest cells differentiate into melanocytes that migrate to the epidermis [36]. Another possible strategy for obtaining a direct experimental proof is to conduct a reporter assay in which the predicted melanocyte-specific enhancer is cloned upstream of a reporter gene or to employ a genome editing technique to disrupt the predicted melanocyte-specific enhancer in the wild type to see if a similar phenotype, namely, a head spot, is observed. Yet another possibility, albeit one that would not provide a direct proof, is to search for other strains or species with a similar phenotype having a mutation in the corresponding genomic region of the predicted enhancer. This would provide strong supporting evidence for the involvement of the predicted enhancer in the development of melanocytes.

Mutations in coding regions may have severe phenotypic effects such as embryonic lethality because, for example, a non-synonymous substitution can lead to the production of proteins without activity or a nonsense mutation can be degraded by nonsense-mediated mRNA decay or produces a truncated protein product. On the other hand, mutations in non-coding regulatory regions, although these have not been studied well compared with those in coding regions, might have milder effects because such mutations sometimes change only the spatio-temporal expression patterns in a certain tissue or in a developmental stage. As we show in this study, the head spot phenotype in the KFRS4/Kyo rat strain is one such phenotype because *Ednrb*-null mutants usually possess both white spots and megacolon, which is a characteristic phenotype of WS4 due to the absence of enteric neurons. Phenotypic effects of the mutations in CNSs are not well documented so far compared with those in protein coding regions, while the fact that the total length of CNSs in a mammalian genome is almost comparable to that of exons [13] leads us to speculate that there must be a number of regulatory mutations that affect various phenotypes. The TargetEC method [13], from which the data used in this study were obtained, can be efficiently applied to identify such regulatory mutations.

Although it is rather straightforward to infer the functional consequence of mutations in gene bodies, it is often difficult to interpret the effect of regulatory mutations, especially if they are located far from their putative target genes. As shown in this study, the efficient use of information about the epigenetic status of genomes, as well as comparative genomic analysis, helps to confirm the functional implications of such regulatory mutations. Epigenomic information, which is rapidly expanding with the progress of several international projects [14, 15], together with information about the three-dimensional conformation of chromatin [16], will shed more light on the detailed regulatory networks of genes.

## Conclusions

We successfully identified a candidate mutation by using the data obtained from our recently devised method, TargetEC, which can detect mutations not only in exons but also in CNSs [13], together with comparative genomic analysis of publicly available epigenomic data. Based on the success rate of exome sequencing studies [37] and the proportion of CNSs, which are thought to be conserved because of their functional importance, there might be a significant number of mutations causative of certain genetic disorders in regulatory regions. By systematically identifying these mutations in genetic disorders, we will be able to deepen our understanding of the regulatory mechanisms of gene expression and disease etiology, which should lead to the development of mechanism-based therapies.

## Additional files

Additional file 1: Table S1. A list of KFRS4/Kyo-specific mutations found within the genomic interval identified by a linkage analysis (chr15:89.0–94.8 Mb in the rn5 assembly). (XLS 36 kb)

Additional file 2: Figure S1. PCR experiments to confirm the deletion found in KFRS4/Kyo. (PDF 68 kb)

Additional file 3: Figure S2. A KFRS4/Kyo-specific deletion of approximately 50 kb in length located approximately 50 kb upstream of *Ednrb*. (PDF 116 kb)

Additional file 4: Figure S3. Virtual 4C output for the region around *Ednrb* gene in mouse genome (mm9 assembly). (PDF 308 kb)

## Abbreviations

CNS: Conserved non-coding sequence
SNV: Single nucleotide variant
WS4: Waardenburg syndrome type 4

## References

[1] Kuramoto T, Yokoe M, Yagasaki K, Kawaguchi T, Kumafuji K, Serikawa T (2010). Genetic analyses of fancy rat-derived mutations. Exp Anim.

[2] Quina LA, Kuramoto T, Luquetti DV, Cox TC, Serikawa T, Turner EE (2012). Deletion of a conserved regulatory element required for Hmx1 expression in craniofacial mesenchyme in the dumbo rat: a newly identified cause of congenital ear malformation. Dis Model Mech.

[3] Reissmann M, Ludwig A (2013). Pleiotropic effects of coat colour-associated mutations in humans, mice and other mammals. Semin Cell Dev Biol.

[4] Puffenberger EG, Hosoda K, Washington SS, Nakao K, de Wit D, Yanagisawa M (1994). A missense mutation of the endothelin-B receptor gene in multigenic Hirschsprung’s disease. Cell.

[5] Gariepy CE, Cass DT, Yanagisawa M (1996). Null mutation of endothelin receptor type B gene in spotting lethal rats causes aganglionic megacolon and white coat color. Proc Natl Acad Sci U S A.

[6] Kunieda T, Kumagai T, Tsuji T, Ozaki T, Karaki H, Ikadai H (1996). A mutation in endothelin-B receptor gene causes myenteric aganglionosis and coat color spotting in rats. DNA Res.

[7] Ceccherini I, Zhang AL, Matera I, Yang G, Devoto M, Romeo G (1995). Interstitial deletion of the endothelin-B receptor gene in the spotting lethal (sl) rat. Hum Mol Genet.

[8] Hosoda K, Hammer RE, Richardson JA, Baynash AG, Cheung JC, Giaid A (1994). Targeted and natural (piebald-lethal) mutations of endothelin-B receptor gene produce megacolon associated with spotted coat color in mice. Cell.

[9] Metallinos DL, Bowling AT, Rine J (1998). A missense mutation in the endothelin-B receptor gene is associated with Lethal White Foal Syndrome: an equine version of Hirschsprung disease. Mamm Genome.

[10] Miwa M, Inoue-Murayama M, Aoki H, Kunisada T, Hiragaki T, Mizutani M (2007). Endothelin receptor B2 (EDNRB2) is associated with the panda plumage colour mutation in Japanese quail. Anim Genet.

[11] Ng SB, Buckingham KJ, Lee C, Bigham AW, Tabor HK, Dent KM (2010). Exome sequencing identifies the cause of a mendelian disorder. Nat Genet.

[12] Rabbani B, Tekin M, Mahdieh N (2014). The promise of whole-exome sequencing in medical genetics. J Hum Genet.

[13] Yoshihara M, Saito D, Sato T, Ohara O, Kuramoto T, Suyama M (2016). Design and application of a target capture sequencing of exons and conserved non-coding sequences for the rat. BMC Genomics.

[14] ENCODE Project Consortium (2012). An integrated encyclopedia of DNA elements in the human genome. Nature.

[15] Kundaje A, Meuleman W, Ernst J, Bilenky M, Yen A, Roadmap Epigenomics Consortium (2015). Integrative analysis of 111 reference human epigenomes. Nature.

[16] Rao SSP, Huntley MH, Durand NC, Stamenova EK, Bochkov ID, Robinson JT (2014). A 3D map of the human genome at kilobase resolution reveals principles of chromatin looping. Cell.

[17] Sato T, Suyama M (2015). ChromContact: a web tool for analyzing spatial contact of chromosomes from Hi-C data. BMC Genomics.

[18] Yoshihara M, Sato T, Saito D, Ohara O, Kuramoto T, Suyama M (2016). Application of target capture sequencing of exons and conserved non-coding sequences to 20 inbred rat strains. Genomics Data.

[19] Speir ML, Zweig AS, Rosenbloom KR, Raney BJ, Paten B, Nejad P (2016). The UCSC Genome Browser database: 2016 update. Nucleic Acids Res.

[20] Li H, Durbin R (2009). Fast and accurate short read alignment with Burrows-Wheeler transform. Bioinformatics.

[21] Li H, Handsaker B, Wysoker A, Fennell T, Ruan J, Homer N (2009). The sequence alignment/map format and SAMtools. Bioinformatics.

[22] McKenna A, Hanna M, Banks E, Sivachenko A, Cibulskis K, Kernytsky A (2010). The genome analysis toolkit: a MapReduce framework for analyzing next-generation DNA sequencing data. Genome Res.

[23] Wang K, Li M, Hakonarson H (2010). ANNOVAR: functional annotation of genetic variants from high-throughput sequencing data. Nucleic Acids Res.

[24] Thorvaldsdóttir H, Robinson JT, Mesirov JP (2013). Integrative genomics viewer (IGV): high-performance genomics data visualization and exploration. Brief Bioinform.

[25] Feng J, Liu T, Qin B, Zhang Y, Liu XS (2012). Identifying ChIP-seq enrichment using MACS. Nat Protoc.

[26] Rintisch C, Heinig M, Bauerfeind A, Schafer S, Mieth C, Patone G (2014). Natural variation of histone modification and its impact on gene expression in the rat genome. Genome Res.

[27] Xiao S, Xie D, Cao X, Yu P, Xing X, Chen C-C (2012). Comparative epigenomic annotation of regulatory DNA. Cell.

[28] Hinrichs AS, Karolchik D, Baertsch R, Barber GP, Bejerano G, Clawson H (2006). The UCSC genome browser database: update 2006. Nucleic Acids Res.

[29] Lieberman-Aiden E, van Berkum NL, Williams L, Imakaev M, Ragoczy T, Telling A (2009). Comprehensive mapping of long-range interactions reveals folding principles of the human genome. Science.

[30] Dixon JR, Selvaraj S, Yue F, Kim A, Li Y, Shen Y (2012). Topological domains in mammalian genomes identified by analysis of chromatin interactions. Nature.

[31] Vietri Rudan M, Barrington C, Henderson S, Ernst C, Odom DT, Tanay A (2015). Comparative Hi-C reveals that CTCF underlies evolution of chromosomal domain architecture. Cell Rep.

[32] Thurman RE, Rynes E, Humbert R, Vierstra J, Maurano MT, Haugen E (2012). The accessible chromatin landscape of the human genome. Nature.

[33] Zhu L, Lee H-O, Jordan CS, Cantrell VA, Southard-Smith EM, Shin MK (2004). Spatiotemporal regulation of endothelin receptor-B by SOX10 in neural crest-derived enteric neuron precursors. Nat Genet.

[34] Hakami RM, Hou L, Baxter LL, Loftus SK, Southard-Smith EM, Incao A (2006). Genetic evidence does not support direct regulation of EDNRB by SOX10 in migratory neural crest and the melanocyte lineage. Mech Dev.

[35] Saldana-Caboverde A, Kos L (2010). Roles of endothelin signaling in melanocyte development and melanoma. Pigment Cell Melanoma Res.

[36] Lee H-O, Levorse JM, Shin MK (2003). The endothelin receptor-B is required for the migration of neural crest-derived melanocyte and enteric neuron precursors. Dev Biol.

[37] Fairfield H, Srivastava A, Ananda G, Liu R, Kircher M, Lakshminarayana A (2015). Exome sequencing reveals pathogenic mutations in 91 strains of mice with Mendelian disorders. Genome Res.

