# Supplementary material for: A deletion in the intergenic region upstream of Ednrb causes head spot in the rat strain KFRS4/Kyo
Source: BMC Genet. 2017 Mar 29;18:29. doi: 10.1186/s12863-017-0497-3 (PMC5372274; doi:10.1186/s12863-017-0497-3)
Supplement: Supplementary file 2 — PCR experiments to confirm the deletion found in KFRS4/Kyo. (PDF 68 kb) [file 12863_2017_497_MOESM2_ESM.pdf]

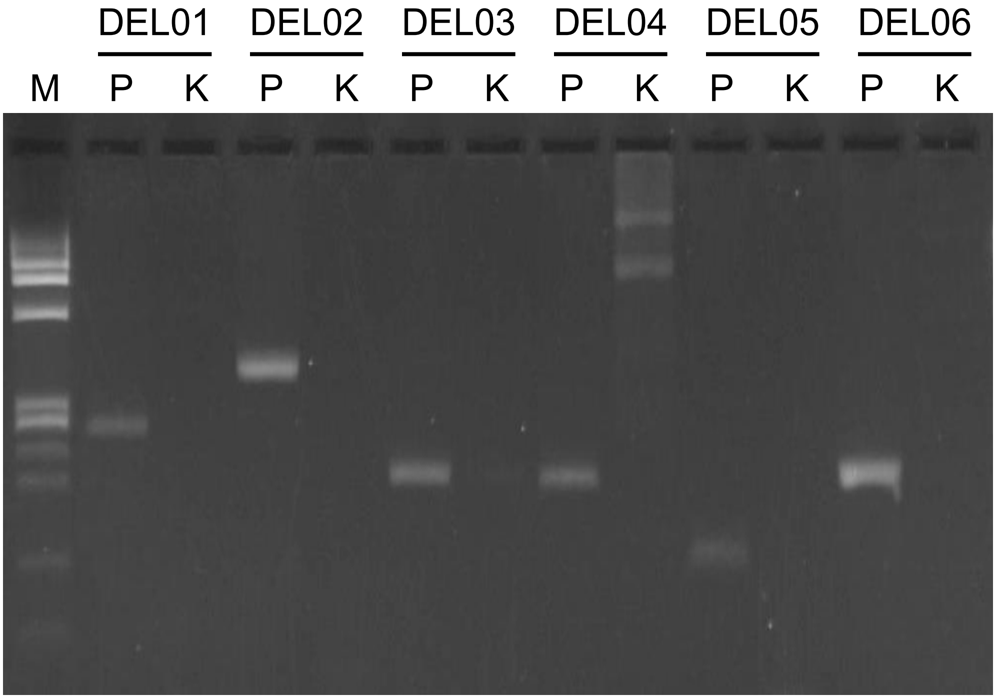

M: molecular marker, ΦX174-HaeIII digest
P: PVG/Seac
K: KFRS4/Kyo

Figure S1. PCR experiments to confirm the deletion found in KFRS4/Kyo. Six regions, each correspond to the deleted CNS. PVG/Seac is used as a control.

Primer sequences

DEL01	F: 5′-TGTCTCATGAGTGTCTGTTTGGA-3′
	R: 5′-TGCTGGCTCTTTGGGAATTCA-3′
DEL02	F: 5′-GGGGTCTGAGGAGGCTCTAA-3′
	R: 5′-TGTGGTAGCTTCTGGTGCTG-3′
DEL03	F: 5′-CCGCGATCCCTCTTTGTGAT-3′
	R: 5′-ACGTGGCTATGCTGTGGAAA-3′
DEL04	F: 5′-GGAATGGAGGGAAGGAGGGA-3′
	R: 5′-ACTACGGCCTTTCCAGTGTG-3′
DEL05	F: 5′-AGTCATTCTGTCCGTGAAAGTCA-3′
	R: 5′-TGAGATATTTGGTAAGGTGGAATTGA-3′
DEL06	F: 5′-CAGTCCTCCTGCCAGACATG-3′
	R: 5′-CAGTGCAGACTCATCCCACA-3′
