# Supplementary material for: A deletion in the intergenic region upstream of Ednrb causes head spot in the rat strain KFRS4/Kyo
Source: BMC Genet. 2017 Mar 29;18:29. doi: 10.1186/s12863-017-0497-3 (PMC5372274; doi:10.1186/s12863-017-0497-3)
Supplement: Supplementary file 3 — A KFRS4/Kyo-specific deletion of approximately 50 kb in length located approximately 50 kb upstream of Ednrb. (PDF 116 kb) [file 12863_2017_497_MOESM3_ESM.pdf]

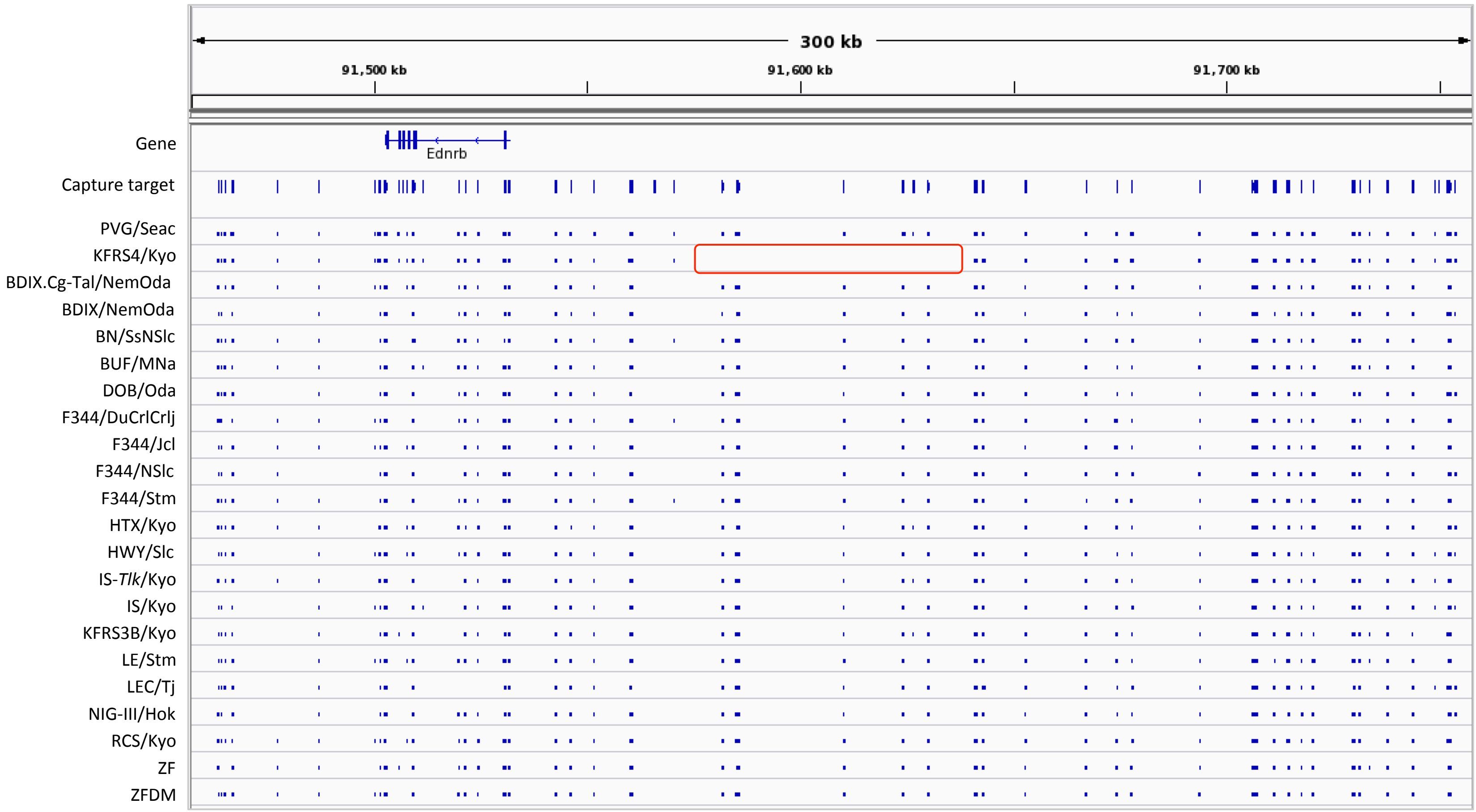

Figure S2. A KFRS4/Kyo-specific deletion of approximately 50 kb in length located approximately 50 kb upstream of *Ednrb*. RefSeq gene and the capture targets of the TargetEC method are shown in the first and the second tracks, respectively. The results of the peak-calling for PVG/Seac and KFRS4/Kyo are shown in the third and fourth tracks, respectively, followed by the results for the peak-calling for the other 20 strains. The KFRS4/Kyo-specific deletion is indicated by a red rectangle.
