# Supplementary material for: A deletion in the intergenic region upstream of Ednrb causes head spot in the rat strain KFRS4/Kyo
Source: BMC Genet. 2017 Mar 29;18:29. doi: 10.1186/s12863-017-0497-3 (PMC5372274; doi:10.1186/s12863-017-0497-3)
Supplement: Supplementary file 4 — Virtual 4C output for the region around Ednrb gene in mouse genome (mm9 assembly). (PDF 308 kb) [file 12863_2017_497_MOESM4_ESM.pdf]

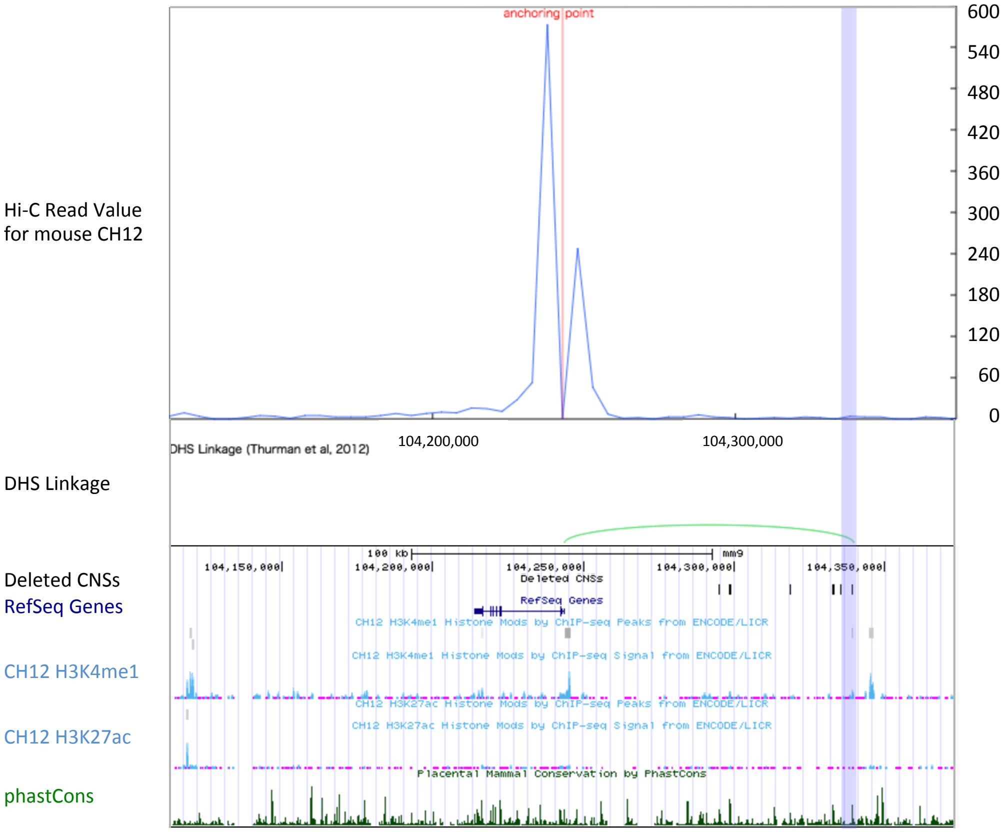

Figure S3. Virtual 4C output for the region around *Ednrb* gene in mouse genome (mm9 assembly). The region containing the predicted enhancer is highlighted in light blue. The top panel shows Hi-C Read Value generated by the Virtual 4C program (http://promoter.bx.psu.edu/hi-c/virtual4c.php) for mouse CH12 cell line. The middle panel shows DHS Linkage [32]. The DHS Linkage (green arc) indicates that the predicted enhancer might interact with the promoter of *Ednrb*. The bottom panel shows the UCSC Genome Browser output for this region. Two histone modification tracks, H3K4me1 and H3K27ac, for CH12 are shown.
